# Effect of anti-inflammatory treatment on systemic inflammation, immune function, and endometrial health in postpartum dairy cows

**DOI:** 10.1038/s41598-020-62103-x

**Published:** 2020-03-23

**Authors:** O. Bogado Pascottini, S. J. Van Schyndel, J. F. W. Spricigo, M. R. Carvalho, B. Mion, E. S. Ribeiro, S. J. LeBlanc

**Affiliations:** 1 Department of Population Medicine, University of Guelph, Guelph, Ontario N1G 2W1 Canada; 2 Department of Animal Biosciences, University of Guelph, Guelph, ON N1G 2W1 Canada

**Keywords:** Medical research, Translational research, Metabolic disorders, Diseases, Reproductive disorders

## Abstract

Systemic inflammation (SI) is increasingly studied in several species because it may be central in many metabolic disturbances and be a risk factor for clinical disease. This proof-of-concept study evaluated the effects of the anti-inflammatory drug meloxicam on markers of SI and energy metabolism, polymorphonuclear neutrophil (PMN) function, and endometritis in clinically healthy postpartum dairy cows. Cows received meloxicam (0.5 mg/kg of body weight; n = 20) once daily for 4 days (10–13 days postpartum) or were untreated (n = 22). Blood samples were collected −7, 1, 3, 5, 7, 10, 11, 12, 13, 14, 18, 21, 28, and 35 days relative to calving to measure serum concentrations of metabolic and inflammatory markers. Function of peripheral blood PMN were evaluated at 5, 10, 14, and 21, and proportion of PMN in endometrial cytology were performed at 5, 10, 14, 21, 28 and 35 days postpartum. Meloxicam decreased serum haptoglobin from the second until the last day of treatment, and improved indicators of energy metabolism (lesser β-hydroxybutyrate and greater insulin-like growth factor-1 during treatment, and greater glucose at the end of treatment than control cows). This improved PMN function at 14 days postpartum, but the endometrial inflammatory status was not affected.

## Introduction

The metabolic profile in obese humans and humans with metabolic syndrome is commonly characterized by upregulated lipolysis, insulin resistance, and systemic inflammation (SI)^1^. The latter are risk factors for type 2 diabetes and cardiovascular disease and are associated with reproductive tract dysfunction^2^. Similarly, the early postpartum period for a lactating dairy cow is a challenging time with increased risk of metabolic and infectious diseases including metritis and endometritis^3^. Around parturition, cows concurrently have a decrease in feed dry matter intake (DMI) and an increase in SI, although it is unclear if one is causative for the other^4^. Postpartum SI is caused by altered metabolism, with several probable contributing causes, including excessive fat mobilization which leads to the production of pro-inflammatory cytokines, tumor necrosis factor alpha (TNF)-α, and interleukin (IL)-6^5,6^. While peripheral insulin insensitivity is an adaptative change to support lactation, these cytokines inhibit intracellular signaling of insulin, heightening insulin insensitivity and exacerbating the release of non-esterified fatty acids (NEFA) from adipose tissue^7^. Proinflammatory cytokines also stimulate the production of acute phase proteins such as haptoglobin, which is commonly used as a biomarker of inflammation^8–10^. Elevated concentrations of circulating NEFA, TNF-α, IL-6 accompanied by insulin resistance resemble sterile inflammation and metabolic syndrome in obese humans and humans with non-alcoholic fatty liver disease^11,12^. In dairy cattle, this metabolic change is mainly due to the homeorhetic shift to support milk production, when nutrient demand exceeds dietary intake, resulting in a state of negative energy balance (NEB)^13^. Several studies have highlighted associations between biomarkers of metabolic stress and inflammation, and reproductive tract disease in dairy cows^14–16^.

In dairy cows, a robust but well-regulated postpartum uterine inflammatory response is necessary for the elimination of pathogens, resumption of ovarian function, and avoidance of reproductive disease^17,18^. Rapid postpartum recruitment of a high number of polymorphonuclear leukocytes (PMN; neutrophils) to the uterine lumen is associated with better uterine health and reproductive performance^19,20^. However, metabolic disorders and elevated circulating concentrations of pro-inflammatory cytokines can lead to compromised immune cell function and dysregulation of inflammation in the uterus^21^. Endometritis (˃ 5% PMN in endometrial cytology diagnosed around the fifth week postpartum) affects 15 to 35% of dairy cows and substantially impairs their fertility^22,23^. Endometritis is chronic, localized, often without concurrent bacterial infection, and so is currently understood as a manifestation of dysregulated inflammation, the specific causes of which are not well characterized^18,24^. Elevated levels of NEFA compromise PMN proliferation and function^25–27^. Cows that subsequently developed endometritis had elevated levels of NEFA and β-hydroxybutyrate (BHB) in the first three weeks postpartum^16^. Compared with healthy cows, those diagnosed with metritis or endometritis were found to have higher serum concentrations of haptoglobin^8^, TNF-α, IL-1-β, and IL-6 and lower concentrations of insulin and IGF-1, preceding the diagnosis of disease^15^.

The use of non-steroidal anti-inflammatory drugs (NSAID) in humans reduced SI associated with insulin resistance and the risk of colon and rectal cancer by inhibiting the cyclooxygenase-2 (COX-2) pathway, tumor formation, and tumor growth^11^. Studies have also shown a reduction in fasting plasma glucose, total cholesterol, triglycerides, and insulin clearance in humans with type 2 diabetes administered high-dose NSAID treatments^28^. In grazing dairy cows, Priest *et al*.^29^ investigated the effects of postpartum NSAID treatment (3 injections at intervals of 3 days) between 21 and 31 days postpartum on endometritis (≥14% PMN in endometrial cytology, diagnosed at 14 days postpartum). They found no effect of treatment on uterine PMN percentage, postpartum anovulatory interval, or milk production, but documented increased pregnancy rate in high-PMN cows that were treated with carprofen. In a study comparing administration of oral sodium salicylate, meloxicam, or placebo, whole-lactation milk and protein yields were greater in cows treated with NSAID^30^. Similarly, cows treated with oral meloxicam produced more milk in early lactation, had reduced odds of subclinical mastitis, and reduced odds of early lactation culling^31^. In a recent study, Montgomery *et al*.^32^ explored the mechanistic role of inflammatory signaling in glucose and energy metabolism of dairy cows treated with sodium salicylate in drinking water in the first week postpartum. Although treated mature transition cows tended to suppress whole-body glucose turnover, no major differences were found in markers of SI and energy metabolism. Meloxicam, a NSAID approved for use in dairy cattle in Canada, selectively inhibits COX-2. However, there is limited research exploring the use of meloxicam to mitigate postpartum SI and treat reproductive tract inflammatory dysregulation in dairy cattle.

The objective of this proof-of-concept experiment was to assess the effects of NSAID treatment on the dynamics of SI, energy metabolism, innate immune response, and uterine health. The hypothesis was that meloxicam would decrease SI, thus improving PMN function and decreasing reproductive tract inflammation assessed by endometrial cytology.

## Materials and Methods

### Study design

This non-randomized controlled trial was conducted from April to August 2018 at the University of Guelph Livestock Research and Innovation Centre, Dairy Facility (Elora, ON, Canada). Animal procedures were approved by the University of Guelph Animal Care Committee, and cows were managed according to the guidelines set by the Dairy Farmers of Canada and the National Farm Animal Care Council. The sample size calculation was based on the effect of anti-inflammatory treatment using 80% power and a 95% confidence level. The sample size of 20 cows per group was based on detection of differences of 0.5 ± 0.5 g/L (mean ± SD) in serum haptoglobin or 10 ± 10% points of PMN in endometrial cytology samples. Only cows considered clinically healthy from calving to 35 days postpartum were included (having unassisted calving, and no retained placenta, metritis or other clinical disease before or during the study period). Initially, 59 non-lactating pregnant Holstein cows (24 nulliparous and 35 parous) were enrolled 14 days before the expected calving date (266 days after the last insemination). Seventeen cows were excluded from the experiment due to injury in the prepartum period (n = 1), dystocia (n = 7), retained placenta (n = 3), or clinical disease (n = 6; metritis, displaced abomasum, or ketosis). The final sample consisted of 42 healthy Holstein cows (17 nulliparous and 25 parous; parity 1.3 ± 1.3), with an average body condition score (BCS) at inclusion of 3.8 ± 0.2 out of 5. Dry cows were housed in free-stall pens and moved to individual calving pens 48 hours before expected calving, or if presenting external signs of preparation for calving (e.g., swelling of the vulva and relaxation of the pelvic ligaments). Five days after calving, healthy cows were relocated to a free-stall lactating pen, where they remained until the end of the experiment (35 days postpartum). In the free-stall pens, daily feed DMI was measured by individual automated feed bins (Insentec B.V., Marknesse, Netherlands). In the individual maternity pen, the amount of offered and refused feed was weighed daily by trained farm personnel. The diets and chemical composition of the lactating cow total mixed ration are described in Supplementary Table A. Cows were fed once daily (1300 h) for *ad libitum* intake after cleaning out of each feed bin. Milking was carried out twice daily (0500 and 1700 h) in a rotary parlour. At inclusion, cows were assigned to one of two experimental treatment groups to deliberately balance for parity and BCS. Experimental groups consisted of control (CON, n = 22; parity 1.3 ± 1.7 and BCS 3.8 ± 0.2) and meloxicam (MEL, n = 20; parity 1.2 ± 1.3 and BCS 3.9 ± 0.2) treatments. MEL received subcutaneous injections of meloxicam (0.5 mg/kg of body weight; Metacam, Boehringer Ingelheim Canada Ltd., Burlington, ON, Canada) once daily on 10, 11, 12 and 13 days postpartum (between 1000 and 1100).

### Blood samples

Blood samples were collected by coccygeal venipuncture into vacuum serum tubes free of anticoagulant (BD Vacutainer Precision Glide, Becton Dickinson, Franklin Lakes, NJ) on -7, 1, 3, 5, 7, 10, 11, 12, 13, 14, 18, 21, 28 and 35 days relative to calving (between 0800 and 0900 h). Within 2 hours of collection, samples were centrifuged at 1,500 × *g* for 15 min and serum was stored in aliquots at −20 °C until analysis.

### Glucose tolerance test

A simplified glucose tolerance test (GTT)^33,34^ was performed at 5, 10, and 14 days postpartum (between 0900 and 1000 h with feed withheld 3 h before and during the GTT). Briefly, cows were restrained to allow jugular intravenous infusion of 0.25 mg of dextrose/kg of body weight (dextrose 50%, Vétoquinol Canada Inc., Lavaltrie, QC, Canada) over 2 min. Blood samples were collected immediately before (0 min), and 10, 60 and 80 min after the dextrose infusion. Within 2 hours, GTT blood samples were centrifuged at 1,500 × *g* for 15 min and serum stored at −20 °C until analysis.

### Blood metabolites

Serum samples were analyzed by the University of Guelph Animal Health Laboratory (Guelph, ON, Canada). Albumin, globulin, total protein, urea, gamma-glutamyl transferase (GGT), aspartate aminotransferase (AST), glutamate dehydrogenase (GLDH), total calcium, and glucose serum concentrations were measured using an autochemistry analyzer (Cobas 6000 c 501, Roche Diagnostics, Indianapolis, IN). NEFA and BHB serum concentrations were measured using Randox NEFA and BHB kits (Randox Laboratories, Canada Ltd., Mississauga, ON, Canada). Haptoglobin concentration was measured by the hemoglobin binding capacity method^35,36^. IGF-I serum concentration was measured using a human immunoassay (Quantikine ELISA, R&D Systems, Minneapolis, MN). Serum insulin concentration was determined using a bovine-specific insulin ELISA (Mercodia AB, Uppsala, Sweden). A summary of the assay names, analytical sensitivities, and intra-assay coefficients of variation for each of the serologic variables are presented in Supplementary Table B.

### Neutrophil function

Circulating PMN function assays were performed at 5, 10, 14, and 21 days postpartum. Blood samples were collected (between 0800 and 0900 h) by coccygeal venipuncture into tubes containing acid citrate dextrose (Vacutainer, Becton Dickinson, Franklin Lakes, NJ) and processed within 3 hours of collection. The PMN isolation protocol was performed as described by Pascottini *et al*.^37^. In the final isolation step (sample free of visible hemoglobin) the cell pellet was resuspended in 500 µL of 1 × PBS. A hemocytometer chamber and trypan blue exclusion were used to assess cell concentration and viability, with only samples with >90% PMN viability being accepted. Cytospin slides were prepared and stained (Wright’s), and cells were tested for purity, with only samples containing >80% PMN being accepted. Samples were centrifuged and the pelleted PMN were diluted to a concentration of 1 × 10^6^/mL using 1 × concentrated PBS with 10% filtered fetal bovine serum (FBS, Invitrogen, Burlington, ON, Canada).

For the oxidative burst assay^37^, 200 µL of the PMN suspension and 2 µL of 1 mM 2′,7′-dihydro-dichlorofluorescein-diacetate (Molecular Probes, Eugene, OR) were incubated in two flow cytometry tubes for 15 min in the dark at 37 °C under gentle agitation. Then, 200 µL of phorbol myristate acetate (Sigma-Aldrich, St. Louis, MO; 25 ng/mL in PBS/FBS) was added to the treatment tube and 200 µL of PBS (1×) containing 10% FBS was added to the control tube. Tubes were incubated for 15 min at 37 °C under gentle agitation and then placed on ice in darkness until flow cytometry analysis.

For the phagocytosis assay^37^, activated normal cow serum was produced for the whole experiment using serum from 20 healthy lactating Holstein cows. Next, 100 mg of Zymosan A from *Saccharomyces cerevisiae* (Sigma-Aldrich) was added per 10 mL of pooled serum, incubated at 37 °C for 60 min under gentle rotation, centrifuged at 2,500 × *g* for 15 min, filtered, and stored at −80 °C. In a flow cytometry tube, 200 µL of the PMN suspension and 50 µL of thawed bovine serum with Zymosan A were incubated with fluorescently labeled 1-µm beads (TransFluo-Spheres Fluorescent Microspheres, Molecular Probes) in the darkness for 30 min at 37 °C. A negative control consisted of PMN incubated without fluorescent beads. After incubation, 200 µL of flow buffer was added to each tube and placed on ice in darkness until flow cytometry analysis.

For the DQ-ovalbumin assay, to measure proteolytic degradation following endocytosis^37^, two tubes containing 1 × 10^6^ PMN in 120 µL of Dulbecco’s modified Eagle’s medium (Gibco/Thermo Fisher Scientific, Waltham, MA) supplemented with 10% FBS were incubated at 37 °C under gentle agitation for 45 min in the dark. One of the tubes served as control and the other was supplemented with 10 µL of DQ-ovalbumin (10 μg/mL; Thermo Fisher Scientific, Eugene, OR). After incubation, cells were washed with PBS, diluted in 200 μL of flow buffer, allocated to flow cytometry tubes on ice, and protected from light until analysis.

PMN samples were analyzed via flow cytometry (FACSCanto, BD Biosciences). The events threshold was set at 10,000 around the area of interest (PMN population) on cytograms of forward versus side scatter. The fluorescence exhibited in the oxidative burst and DQ-ovalbumin assay were measured at 530 nm on a log scale. Phagocyted fluorescent beads were measured at 780 nm on a log scale. Data were analyzed using FlowJo software (Tree Star, Ashland, OR). A gate was labeled around the PMN population and the cytograms were transformed to histograms. Control and treatment PMN histograms were visualized to corroborate the functionality of the assays. For all the PMN function assays, the difference in the median fluorescence intensity (MFI) was calculated relative to the MFI of the respective negative control. For oxidative burst, the shift in the percentage of stimulated PMN relative to the negative control, and for phagocytosis, the percentage of PMN that engulfed cells, were also evaluated.

### Endometrial cytology

Cytobrush samples were collected at 5, 10, 14, 21 days postpartum, as described by Van Schyndel *et al*.^38^. Briefly, after cleansing the perineal area of the cow, the cytobrush rod (covered with a plastic sanitary sheath) was introduced into the vagina and guided through the cervix via rectal palpation. Once the tip of the rod reached the uterine body, the sanitary sheath was pulled back, the cytobrush was exposed from the rod and rotated against the dorsal wall of the endometrium^39^. The cytobrush was retracted into the rod and removed from the vagina, then rolled onto a microscope slide, air-dried, and stained using May-Grunwald-Giemsa stain. Three hundred cells were counted per slide in multiple fields and the PMN to epithelial cells ratio was averaged between the outcomes of two observers (Lin’s concordance correlation coefficient ρc = 0.77 (0.71–0.82)).

### Statistical analyses

The statistical analyses were performed using R-core (version 3.6.1; R Core Team, Vienna, Austria). Results are expressed as least squares means and standard errors. Variables were analyzed by Shapiro-Wilk’s test and when not normally distributed (*P* < 0.05), were log_10_ transformed. The effect of explanatory variables on dependent variables were fitted in mixed linear regression models. Treatment (CON and MEL), sampling day, and their interaction were forced into each model. Parity (nulliparous and parous) and BCS at enrollment (≤3.5 and ≥3.75) were offered as covariates for all models. Sampling day was included as a repeated observation within the random cow factor. For results from the simplified GTT, the area between insulin and glucose curves was calculated using the trapezoid method^33^ and fitted in mixed linear regression models including parity and BCS as covariates. First-order interactions of treatment with each covariate were tested and excluded when *P* > 0.05. The overall effects of predictor variables (treatment, sampling day, and treatment × sampling day interaction) were assessed with a type III analysis of variance and differences between levels of predictor variables were assessed using Tukey’s post hoc test.

## Results

### DMI and milk production

The daily DMI for CON and MEL groups are presented in Supplemental Fig. 1. No differences in DMI were found between experimental groups at any timepoint (*P* = 0.3). Mean milk production to 35 day postpartum was not different between experimental groups (*P* = 0.8; 33.2 ± 10.6 kg for CON and 33.5 ± 10.7 kg for MEL; Supplemental Fig. 2).

### Blood metabolites

**Figure 1:**
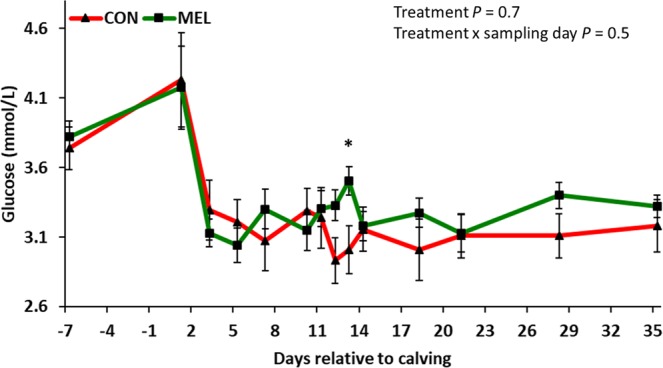
LSM (accounting for parity and body condition score) ± SE of serum glucose concentrations in 42 Holstein cows. Experimental groups consisted of control (CON; n = 22) and meloxicam treated cows (MEL, n = 20). MEL received meloxicam (0.5 mg/kg of body weight) once daily for 4 days (10 to 13 days postpartum). MEL had higher serum glucose concentrations when compared to CON at 13 days postpartum (*P* = 0.04).

**Figure 2:**
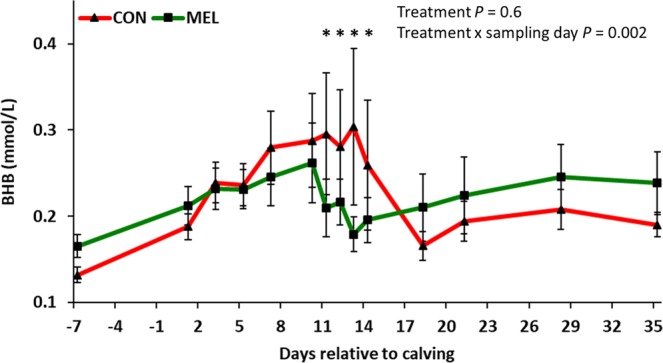
Log_10_-scale LSM (accounting for parity and body condition score) ± SE of serum β-hydroxybutyrate (BHB) concentrations in 42 Holstein cows. Experimental groups consisted of control (CON; n = 22) and meloxicam treated cows (MEL, n = 20). MEL received meloxicam (0.5 mg/kg of body weight) once daily for 4 days (10 to 13 days postpartum). MEL had lower serum BHB concentrations when compared to CON at 11, 12, 13, and 14 days postpartum (*P* = 0.01, 0.04, 0.001, and 0.05, respectively).

**Figure 3:**
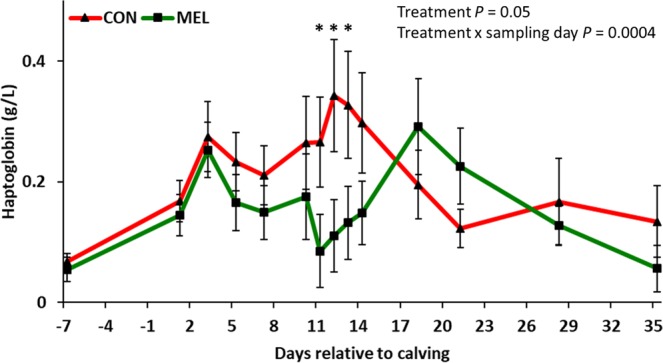
Log_10_-scale LSM (accounting for parity and body condition score) ± SE serum haptoglobin concentrations in 42 Holstein cows. Experimental groups consisted of control (CON; n = 22) and meloxicam treated cows (MEL, n = 20). MEL received meloxicam (0.5 mg/kg of body weight) once daily for 4 days (10 to 13 days postpartum). MEL had lower serum haptoglobin concentrations when compared to CON at 11, 12, and 13 days postpartum (*P* = 0.05, 0.02, and 0.05, respectively).

**Figure 4:**
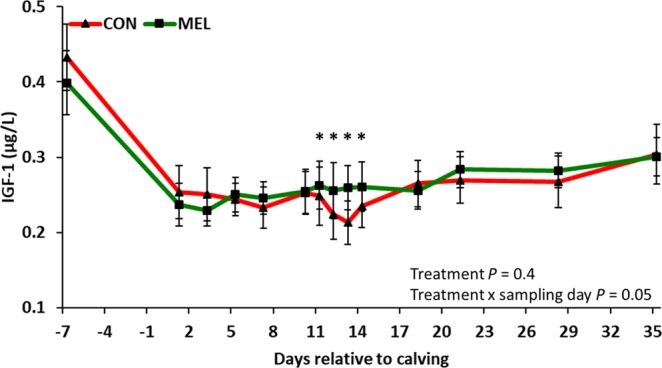
Log_10_-scale LSM (accounting for parity and body condition score) ± SE of serum insulin-like growth factor-1 (IGF-1) concentrations in 42 Holstein cows. Experimental groups consisted of control (CON; n = 22) and meloxicam treated cows (MEL, n = 20). MEL received meloxicam (0.5 mg/kg of body weight) once daily for 4 days (10 to 13 days postpartum). MEL had higher serum IGF-1 concentrations when compared to CON at 11, 12, 13, and 14 days postpartum (*P* = 0.04, 0.002, 0.0003, and 0.007, respectively).

All serum metabolites changed (*P* < 0.05) over time in both experimental groups. MEL had higher serum glucose concentrations than CON at the last day of meloxicam treatment (13 days postpartum; *P* = 0.04; Fig. 1). Although serum NEFA concentrations were similar between experimental groups (*P* = 0.9; Supplemental Fig. 3), serum BHB concentrations were lower in MEL than CON during meloxicam treatment (11, 12, 13, and 14 days postpartum; *P* = 0.01, 0.04, 0.001, and 0.05, respectively; Fig. 2). Similarly, serum haptoglobin concentrations were lower in MEL on 11, 12, and 13 days postpartum (*P* = 0.05, 0.02, and 0.05, respectively; Fig. 3). Serum IGF-1 concentrations were stable in MEL from the first until the last day of treatment but decreased in CON, such that IGF-1 concentrations were greater in the treated cows (11, 12, 13, and 14 days postpartum; *P* = 0.04, 0.002, 0.0003, and 0.007, respectively; Fig. 4). Total calcium, total protein, albumin, globulin, cholesterol, and urea were not different between CON and MEL groups at any time point (*P* > 0.2; Supplemental Figs. 4–9). None of the serologic concentrations of liver enzymes (GGT, AST, and GLDH) were different between experimental groups at any timepoint (*P* > 0.7; Supplemental Figs. 10–12).

### Glucose tolerance test

The response of serum glucose and insulin to dextrose infusion at each time point of the GTT (0, 10, 60, and 80 min) is presented in Supplemental Fig. 13. No treatment effects were found in the area between the insulin and glucose curves in the GTT performed at 5, 10, or 14 days postpartum (*P* = 0.5, 0.2, and 0.5, respectively).

### Neutrophil function

**Figure 5:**
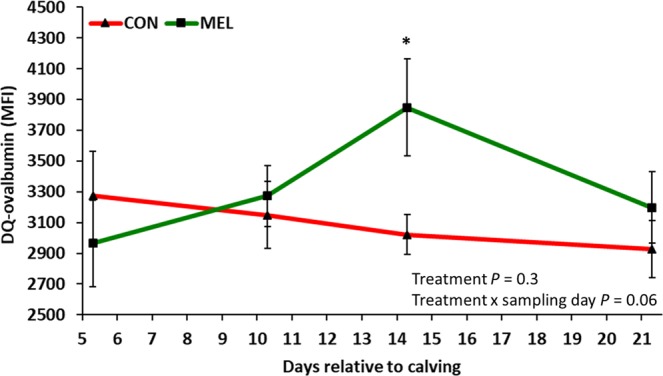
LSM (accounting for parity and body condition score) ± SE of median fluorescence intensity (MFI) of circulating polymorphonucleated neutrophils (PMN) incubated with DQ-ovalbumin in 42 Holstein cows. Experimental groups consisted of control (CON; n = 22) and meloxicam treated cows (MEL, n = 20). MEL received meloxicam (0.5 mg/kg of body weight) once daily for 4 days (10 to 13 days postpartum). MEL had greater DQ-ovalbumin MFI than CON at 14 days postpartum (*P* = 0.008).

For the oxidative burst assay, the MFI and the percentage of PMN with oxidative burst activity were not different between MET and CON at any time point (*P* > 0.9; Supplemental Figs. 14 and 15, respectively). Although the MFI for phagocytosis did not differ between experimental groups (*P* = 0.7; Supplemental Fig. 16), the percentage of PMN that engulfed beads at 21 days postpartum tended to be greater for MEL (*P* = 0.09; Supplemental Fig. 17). For the DQ-ovalbumin assay the MFI was 27% greater in MEL at 14 days postpartum (*P* = 0.008; Fig. 5).

### Endometrial cytology

**Figure 6:**
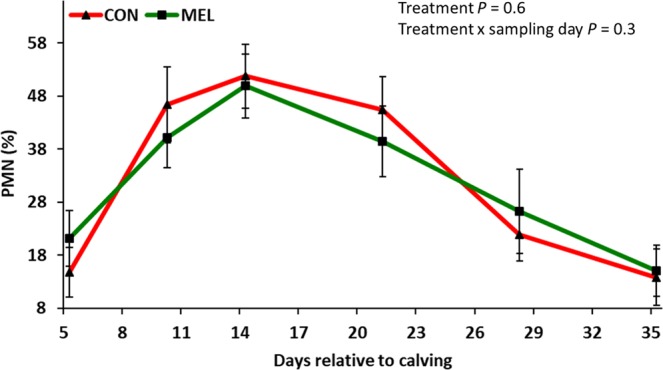
LSM (accounting for parity and body condition score) ± SE of endometrial polymorphonuclear cells percentage (PMN, %) of 42 Holstein cows. Experimental groups consisted of control (CON; n = 22) and meloxicam treated cows (MEL, n = 20). MEL received meloxicam (0.5 mg/kg of body weight) once daily for 4 days (10 to 13 days postpartum). The PMN % was similar between CON and MEL.

The PMN percentage in the endometrial cytology samples changed over time (*P* < 0.05), reaching a peak at 14 days postpartum, decreasing until 35 days postpartum for both treatment groups, but did not differ between treatments (*P* = 0.6; Fig. 6).

No adverse events (apparent clinical abnormalities or disease events) occurred during the study.

## Discussion

This study evaluated the hypothesis that administration of NSAID in the second week after calving could mitigate SI and attenuate endometrial inflammation. Meloxicam treatment decreased serum haptoglobin concentrations (a marker of SI), and improved indicators of adaptative energy metabolism (lesser serum concentrations of BHB, greater glucose and stable IGF-1 in the treatment period). This was associated with modest improvement of one measure of PMN function but did not translate to reduced inflammation of the endometrium.

The foundation of the therapeutic effects of NSAID is through inhibition of COX enzymes, which reduces the formation of prostaglandins^40^. COX exists in two isoforms, the constitutive COX-l and the inducible COX-2^41^. Inhibition of COX-1 accounts for most of the negative side-effects associated with NSAID use, while COX-2 inhibition accounts for the therapeutic effects of NSAIDs^42^. However, a growing body of evidence suggests that NSAID have supplementary anti-inflammatory properties. Treatment with NSAID could modulate transcription factors such as nuclear factor-kappa B^43^, that controls the inducible expression of many genes involved in inflammation, including TNF-α and IL-1β^44^. Such an effect may explain the decreased concentration of serum haptoglobin from the second until the last day of meloxicam treatment. While expected, to our knowledge, this is the first time this effect has been demonstrated in dairy cattle. This may provide new opportunities to mitigate the well-documented detrimental associations of elevated postpartum haptoglobin concentrations with reproductive health in dairy cows^45–47^.

Glucose is the main fuel used by most cells, but in lactating dairy cows a large portion of hepatic glucose output is directed to the mammary gland to support milk production. Alternative substrates are therefore required for energy metabolism in the peripheral tissue of lactating cows. This partitioning of nutrients in the early postpartum period coincides with a decrease in DMI^48^, and an increase of metabolic markers associated with NEB, such as NEFA and BHB^49^. Circulating NEFA concentrations reflect fat mobilization and BHB concentrations reflect the degree of oxidation of fatty acids in the liver^50–52^. Although we found no differences in milk production or DMI between experimental groups during or after meloxicam treatment, BHB concentrations were substantially lower in MEL during treatment. Lower biosynthesis of haptoglobin may improve hepatic metabolism of fatty acids. Glucose and IGF-1 concentrations are important markers of adaptation to NEB^53^. Therefore, our results validate a positive effect of meloxicam treatment on liver function and energy status of postpartum dairy cows, as indicated by decreased serum BHB concentrations and increased serum glucose. Interestingly, serum IGF-1 concentrations were stable during treatment with MEL, but decreased at that time in CON, such that IGF-1 was greater in MEL than CON. The greater concentrations of haptoglobin and BHB during this time in CON might indicate impaired liver health, which could transiently impair IGF-1 synthesis. However, the specific mechanisms behind these results remain to be determined.

Maladaptation to NEB in dairy cows is associated with a decline in PMN function^54–56^. PMN mainly depend on glucose for functions such as chemotaxis and bacterial killing^53,57,58^. High concentrations of NEFA, BHB, and haptoglobin are associated with decreased PMN function^25,27,59^. We observed a modest effect of NSAID treatment on MFI in the DQ-ovalbumin assay 14 days postpartum (the day after the last meloxicam injection). The DQ-ovalbumin assay reflects degradation of ovalbumin after endocytosis and proteolytic digestion^60^. This modest improvement in PMN function may be a result of mitigation of SI and an improved glucose availability for PMN.

Though it is unclear exactly how metabolism and immunity are integrated with endometrial health^61^, there is increased evidence of important roles of SI and liver dysfunction in endometrial inflammation^29,62,63^. A longitudinal study examining the dynamics of endometrial cytology demonstrated that rapid postpartum recruitment of PMN to the uterine lumen was associated with improved fertility^20^. The proportion of PMN in the uterine lumen should reach a peak one to two weeks postpartum and it is important for endometrial health that PMN numbers return to basal levels by the fourth week postpartum^19,20^. Persistently elevated PMN percentage in the endometrium is associated with a substantial reduction in the probability of pregnancy following insemination^64^. While in the current study MEL succeeded in decreasing one measure of SI, improving indicators of energy metabolism, and slightly improving PMN function, this did not translate to a reduction in endometrial inflammation at any time point (14, 21, 28 or 35 days postpartum). In two studies in grazing dairy cows^29,65^, administration of a different NSAID at 1, 3, and 5 or 19, 21, and 23 days postpartum, or 3 injections at intervals of 3 days between 21 and 31 days postpartum, did not have an effect on endometrial health. However, NSAID treatment between 21 and 31 days postpartum increased the pregnancy rate in high-PMN cows. Despite of these improvements in fertility, the authors hypothesized that the lack of effect on the endometrial PMN proportion might be attributable to the timing of treatment and the failure to produce sufficient suppression of inflammation due to the dosing interval. Based on their results, for this study we used a more aggressive treatment regimen for four consecutive days just after the PMN peak as described by Gilbert *et al*.^20^. This timing of treatment is also coincident with the divergent systemic and uterine inflammatory status in cows later diagnosed with endometritis as reviewed by LeBlanc^3^. The PMN percentage in endometrial cytology reached a peak 14 days postpartum regardless of treatment, and then decreased until 35 days postpartum for both experimental groups. Based on the effect of MEL on serum haptoglobin, treatment appears to have had the intended effect of reducing SI, so the results suggest that of inflammation within the endometrium may be localized, or that the changes in indicators of SI with systemic MEL were insufficient to effect changes in uterine inflammation.

No other measured metabolite or insulin sensitivity (measured via GTT) was influenced by meloxicam treatment in this experiment. Arguably, the repeated sampling for measuring endometrial PMN% may have created or aggravated inflammation of the endometrium. However, Madoz *et al*.^66^ did not find an effect of repeated cytobrush sampling (4 cytobrush samples in a 4- to 7-day interval) in lactating cows (27 to 56 days postpartum). To the extent that sampling may have created artefacts in endometrial PMN%, the effect would be equal in both treatment groups. Other studies involving NSAID in the postpartum period appeared to improve liver function, with reductions in plasma AST and GLDH^29^. Moreover, oral anti-inflammatory medication in the first week postpartum resulted in greater hepatic insulin sensitivity^32,67,68^. Other early postpartum studies with NSAID have been performed, but the metabolic effects are not consistent^30^. The type of NSAID, route and frequency of administration, dosage, and inclusion criteria for animals (only healthy versus including diseased), likely contribute to these discrepancies.

## Conclusion

This study used validated, minimally-invasive biomarkers to evaluate the effects of meloxicam treatment on postpartum inflammation, circulating PMN function, and uterine health in dairy cows. Meloxicam treatment attenuated SI (decreased serum haptoglobin concentrations) and improved indicators of energy metabolism (decreased serum concentrations of BHB and increased glucose and IGF-1). This resulted in slight improvement in PMN function, which did not translate to mitigation of inflammation in the endometrium. Further investigation is warranted to explore the mechanisms of action of anti-inflammatory treatments on SI, energy metabolism, and to optimize the drug dosages, timing, and duration to improve animal health.

## Electronic supplementary material

Supplementary information.
