# Supplementary material for: Effect of anti-inflammatory treatment on systemic inflammation, immune function, and endometrial health in postpartum dairy cows
Source: Sci Rep. 2020 Mar 23;10:5236. doi: 10.1038/s41598-020-62103-x (PMC7090035; doi:10.1038/s41598-020-62103-x)
Supplement: Supplementary file 1 — Supplementary information. [file 41598_2020_62103_MOESM1_ESM.docx]

**Effect of anti-inflammatory treatment on systemic inflammation, immune function, and endometrial health in postpartum dairy cows**

O. Bogado Pascottini^1*^, S. J. Van Schyndel^1^, J. F. W. Spricigo^2^, M. R. Carvalho^2^, B. Mion^2^, E. S. Ribeiro^2^ & S. J. LeBlanc^1^

***^1^*** *Department of Population Medicine, University of Guelph, Guelph, Ontario, Canada N1G 2W1*

***^2^*** *Department of Animal Biosciences, University of Guelph, Guelph, ON, Canada N1G 2W1*

^*^Corresponding author: O. Bogado Pascottini.

^*^Current e-mail address: [osvaldo.bogado@ugent.be](mailto:osvaldo.bogado@ugent.be)

| **Supplementary Table A.** Descriptive summary of diet and nutrient analysis for total mixed ration for lactating cows (1 to 35 days postpartum) for 45 Holstein cows^1^ | |
| --- | --- |
| **Item** | **TMR lactating cows** |
| **Ingredient (% of dry matter weight)** |  |
| Haylage | 31.7 |
| Corn silage | 30.3 |
| High-moisture corn | 22.1 |
| Concentrate mix^*^ | 14.1 |
| Straw | 1.8 |
| **^*^Concentrate composition (kg/1,000 kg)** |  |
| Soy Plus^2^ | 400.00 |
| Soybean meal 47% | 300.00 |
| Canola | 100.00 |
| Wheat shorts | 34.00 |
| Sodium sesquicarbonate | 31.00 |
| Limestone calcium carbonate | 29.00 |
| Fine salt | 29.00 |
| Magnesium oxide | 15.5 |
| Yeast | 14.00 |
| Monocalcium phosphate | 14.00 |
| Potassium carbonate | 10.00 |
| FFM Org Ruminant Micro^3^ | 7.00 |
| Urea | 8.00 |
| MetaSmart^4^ | 6.00 |
| Sulphur 99.5% | 2.13 |
| Micro aid concentrate^5^ | 0.33 |
| **Nutrient (% of DM, unless otherwise indicated)^5^** |  |
| DM (%) | 44.9 ± 1.0 |
| Crude Protein | 14.0 ± 0.8 |
| Soluble protein | 4.9 ± 0.3 |
| Acid Detergent Fibre | 16.5 ± 2.0 |
| Neutral Detergent Fibre | 31.3 ± 2.8 |
| Lignin | 2.5 ± 0.4 |
| Fat | 3.0 ± 0.1 |
| Starch | 28.5 ± 4.1 |
| Non-fibre carbohydrate | 48.1 ± 3.5 |
| Ash | 5.7 ± 0.5 |
| Ca | 0.7 ± 0.06 |
| P | 0.3 ± 0.01 |
| K | 1.3 ± 0.12 |
| Mg | 0.3 ± 0.02 |
| Na | 0.3 ± 0.04 |
| Net energy lactation (Mcal/kg of DM) | 1.6 ± 0.03 |
| ^1^Based on the analysis of n = 8 feed samples. Experimental groups consisted of control (CON; n = 22) and meloxicam treated cows (MEL, n = 20). MEL cows received meloxicam (0.5 mg/kg of body weight) once daily for 4 days (10 to 13 days postpartum). There were no differences between experimental groups.^2^Soy plus (Landus cooperative, Ames, IA, USA): bypass soybean meal. ^3^FFM Org Ruminant Micro (Floradale Feed Mill, Floradale, ON, Canada): trace mineral and vitamin premix containing 5 organic microminerals (zinc, manganese, copper, cobalt, and selenium). ^4^MetaSmart (Adisseo, Alpharetta, GA): rumen-protected methionine. ^5^Micro-Aid Feed Grade Concentrate (DPI Global, Porterville, CA, USA): environmentally safe odor-eliminating product. ^5^Values are mean ± SD. | |

| **Supplementary Table B.** Summary of the lower limit of quantification and intra-assay coefficients of variation of each of the serum metabolites analysed in this experiment. Experimental groups consisted of control (CON; n = 22) and meloxicam treated cows (MEL, n = 20). MEL cows received meloxicam (0.5 mg/kg of body weight) once daily for 4 days (10 to 13 days postpartum). Blood samples were collected on -7, 1, 3, 5, 7, 10, 11, 12, 13, 14, 18, 21, 28 and 35 days relative to calving. Within 2 hours of collection, samples were centrifuged at 1,500 × g for 15 min and serum was stored in aliquots at -20°C until analysis. Before analysis, serum samples were thawed at room temperature. | | | |
| --- | --- | --- | --- |
| **Variable** | **Assay** | **Lower limit of quantification** | **Intra-assay coefficient of variation (%)** |
| Total calcium^1,2^ | CA2 | 0.2 mmol/L | 1.7 |
| Total protein^1,2^ | TP2 | 2 g/L | 2.4 |
| Albumin^1,2^ | ALB2 | 2 g/L | 2.5 |
| Globulin^3^ | - | - | - |
| Cholesterol^1,2^ | CHOL2 | 0.1 mmol/L | 3.1 |
| Urea^1,2^ | UREAL | 0.5 mml/L | 3.6 |
| Glucose^1,2^ | GLUC3 | 0.1 mml/L | 1.9 |
| Gamma-glutamyl transferase^1,2^ | GGT2 | 3 U/L | 3.1 |
| Aspartate aminotransferase^1,2^ | ASTL | 1 U/L | 1.9 |
| Glutamate dehydrogenase^1,2^ | GLDH3 | 5 U/L | 3.4 |
| β-hydroxybutyrate^1,4^ | RANBUT | 0.1 mmol/L | 4.9 |
| Non-esterified fatty acids^1,4^ | NEFA | 0.1 mml/L | 4.5 |
| Haptoglobin^1,5^ | - | 0.03 g/L | 4.4 |
| Insulin growth factor-1^6^ | IGF-1 | 0.026 μ/L | 3.2 |
| Insulin^7^ | Insulin | 0.025 μ/L | 3.9 |
| ^1^Samples analysed by Animal Health Laboratory, University of Guelph, for metabolic profile by an autochemistry analyzer (Cobas 6000 c 501, Roche Diagnostics, Indianapolis, IN).  ^2^Roche Diagnostics GmbH, Sandhofer Strasse 116, D-68305 Mannheim, PA.  ^3^Globulin was calculated by the difference between total protein and albumin concentrations.  ^4^Randox Laboratories, Canada Ltd., Mississauga, ON, Canada.  ^5^Measured by the hemoglobin binding capacity method (Makimura and Suzuki, 1982; Skinner et al., 1991).  ^6^Quantikine ELISA, R&D Systems, Minneapolis, MN. Read with a spectrophotometer (EON, Biotek, Winooski, VT).  ^7^Bovine-specific insulin ELISA (Mercodia AB, Uppsala, Sweden). | | | |


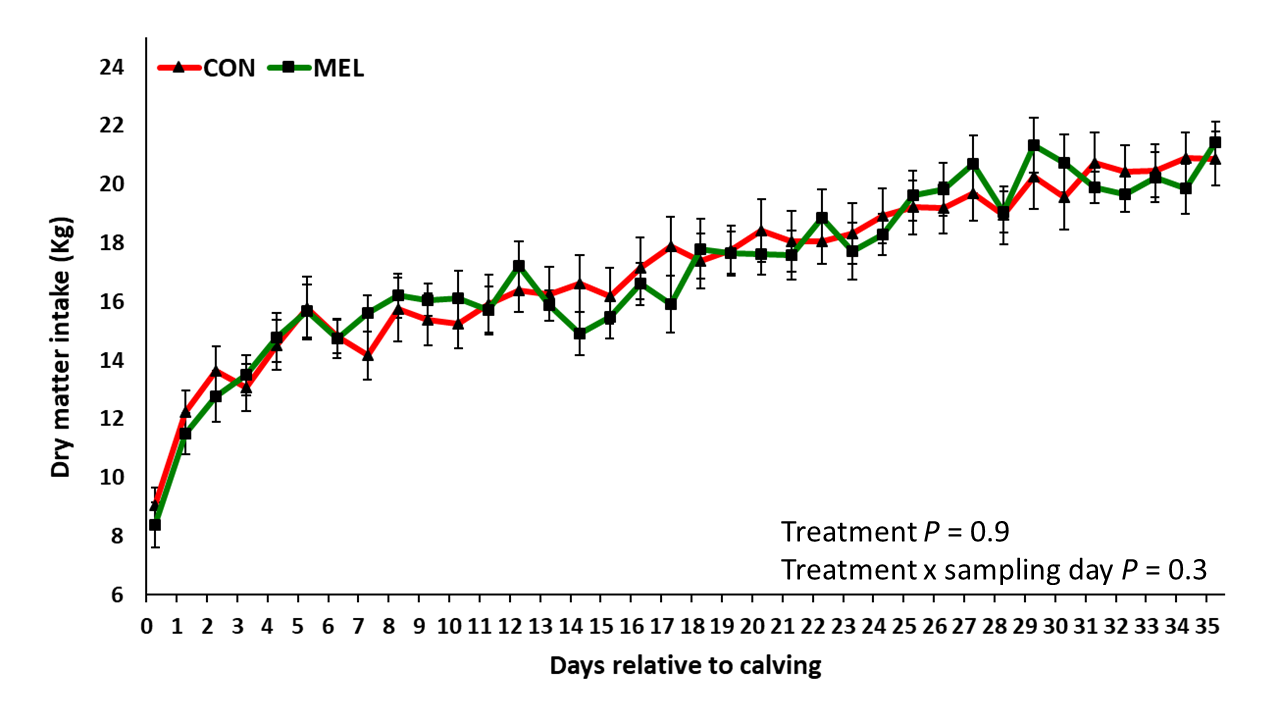


**Supplemental Figure 1.** LSM (accounting for parity and body condition score) ± SE of feed dry matter intake of Holstein cows (n = 42). Experimental groups consisted of control (CON; n = 22) and meloxicam treated cows (MEL, n = 20). MEL received meloxicam (0.5 mg/kg of body weight) once daily for 4 days (10 to 13 days postpartum). There were no differences between experimental groups.


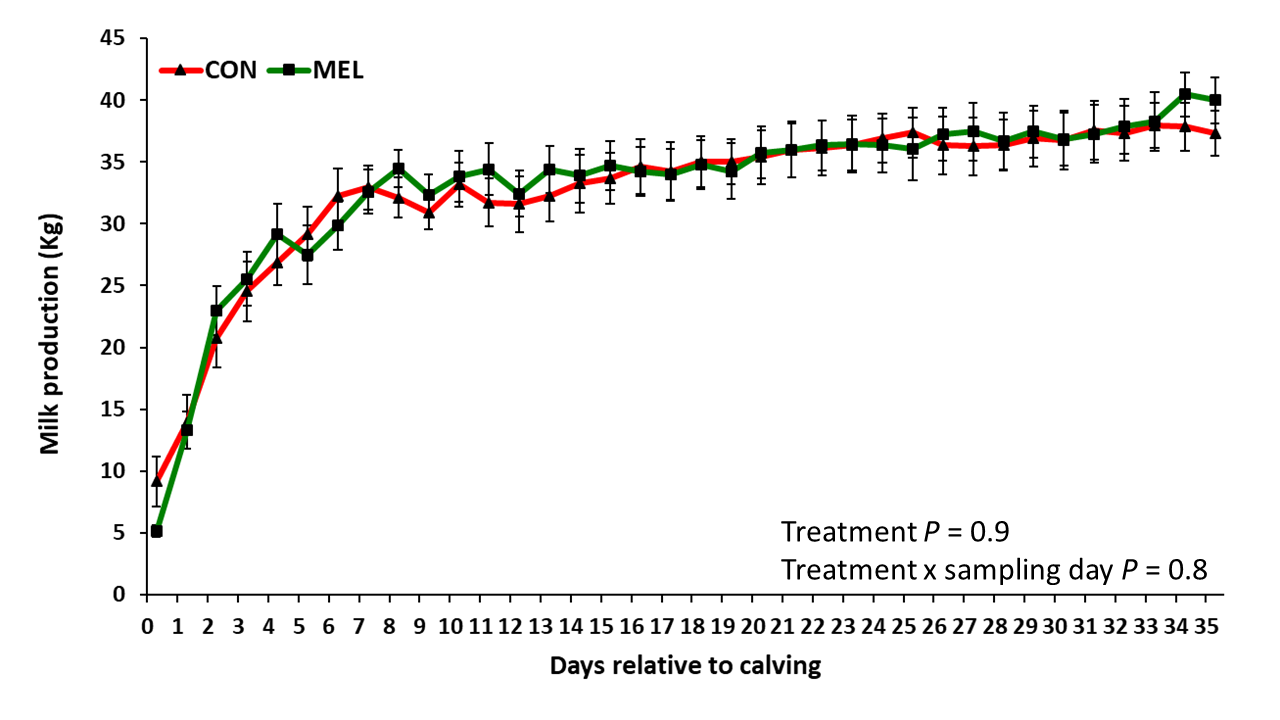


**Supplemental Figure 2.** LSM (accounting for parity and body condition score) ± SE of daily milk production of Holstein cows (n = 42). Experimental groups consisted of control (CON; n = 22) and meloxicam treated cows (MEL, n = 20). MEL received meloxicam (0.5 mg/kg of body weight) once daily for 4 days (10 to 13 days postpartum). There were no differences between experimental groups.


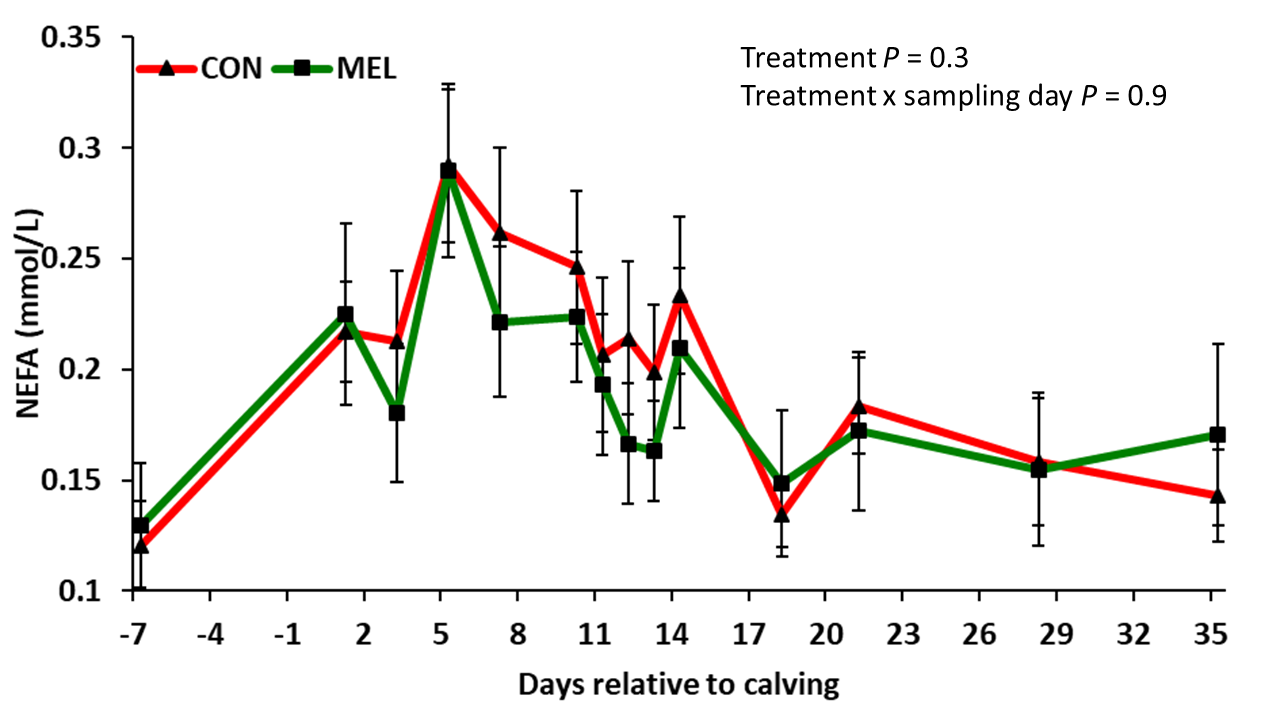


**Supplemental Figure 3.** Log_10_-scale LSM (accounting for parity and body condition score) ± SE of serum non-esterified fatty acids (NEFA) concentrations in 42 Holstein cows. Experimental groups consisted of control (CON; n = 22) and meloxicam treated cows (MEL, n = 20). MEL received meloxicam (0.5 mg/kg of body weight) once daily for 4 days (10 to 13 days postpartum). There were no differences between experimental groups.


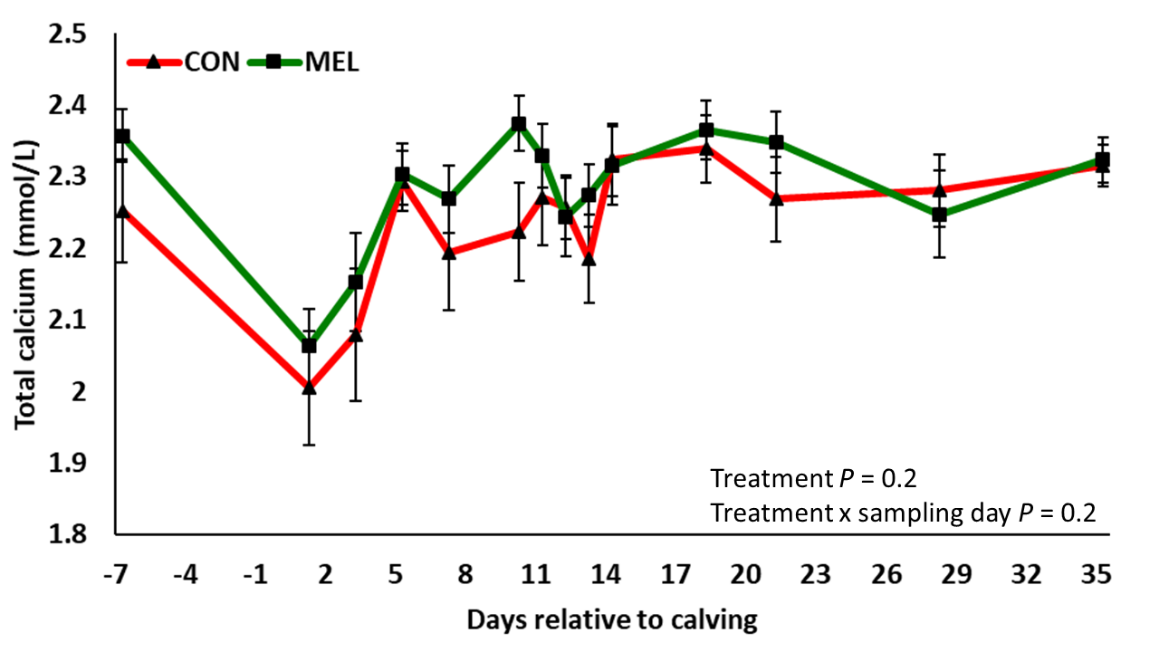


**Supplemental Figure 4.** LSM (accounting for parity and body condition score) ± SE of serum total calcium concentrations in 42 Holstein cows. Experimental groups consisted of control (CON; n = 22) and meloxicam treated cows (MEL, n = 20). MEL received meloxicam (0.5 of mg/kg body weight) once daily for 4 days (10 to 13 days postpartum). There were no differences between experimental groups.


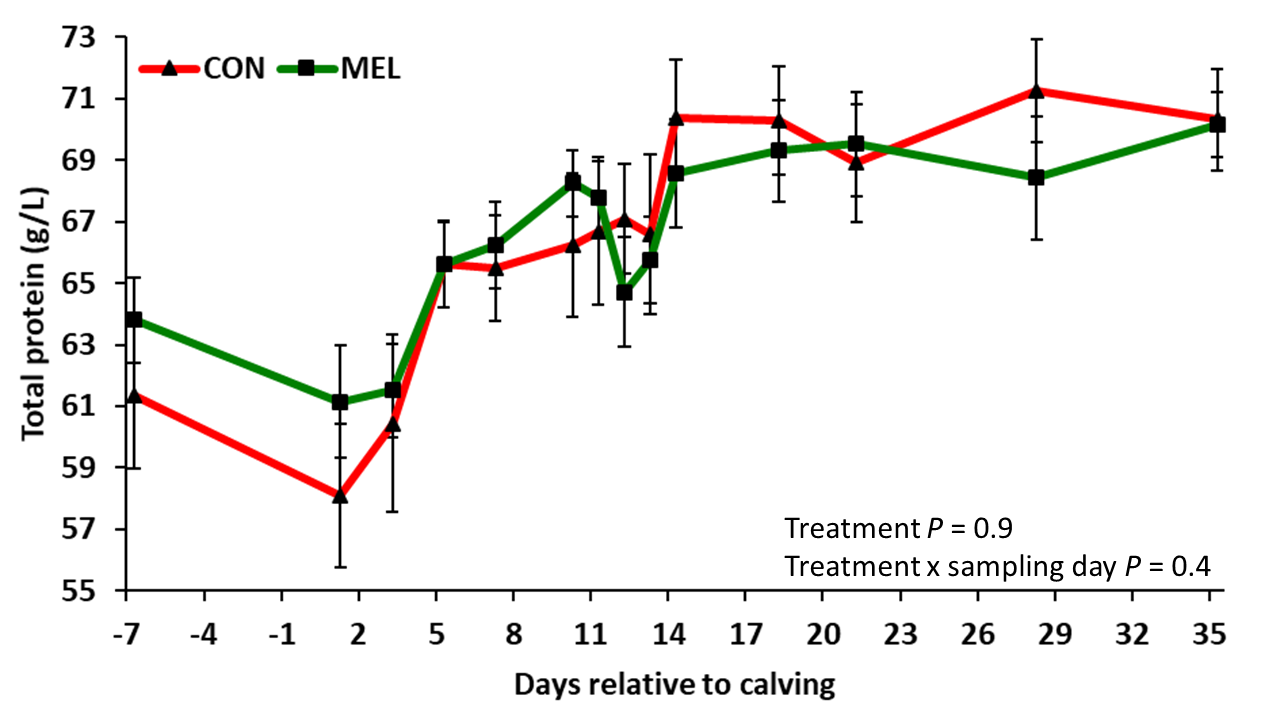


**Supplemental Figure 5.** LSM (accounting for parity and body condition score) ± SE of serum total protein concentrations in 42 Holstein cows. Experimental groups consisted of control (CON; n = 22) and meloxicam treated cows (MEL, n = 20). MEL received meloxicam (0.5 of mg/kg body weight) once daily for 4 days (10 to 13 days postpartum). There were no differences between experimental groups.


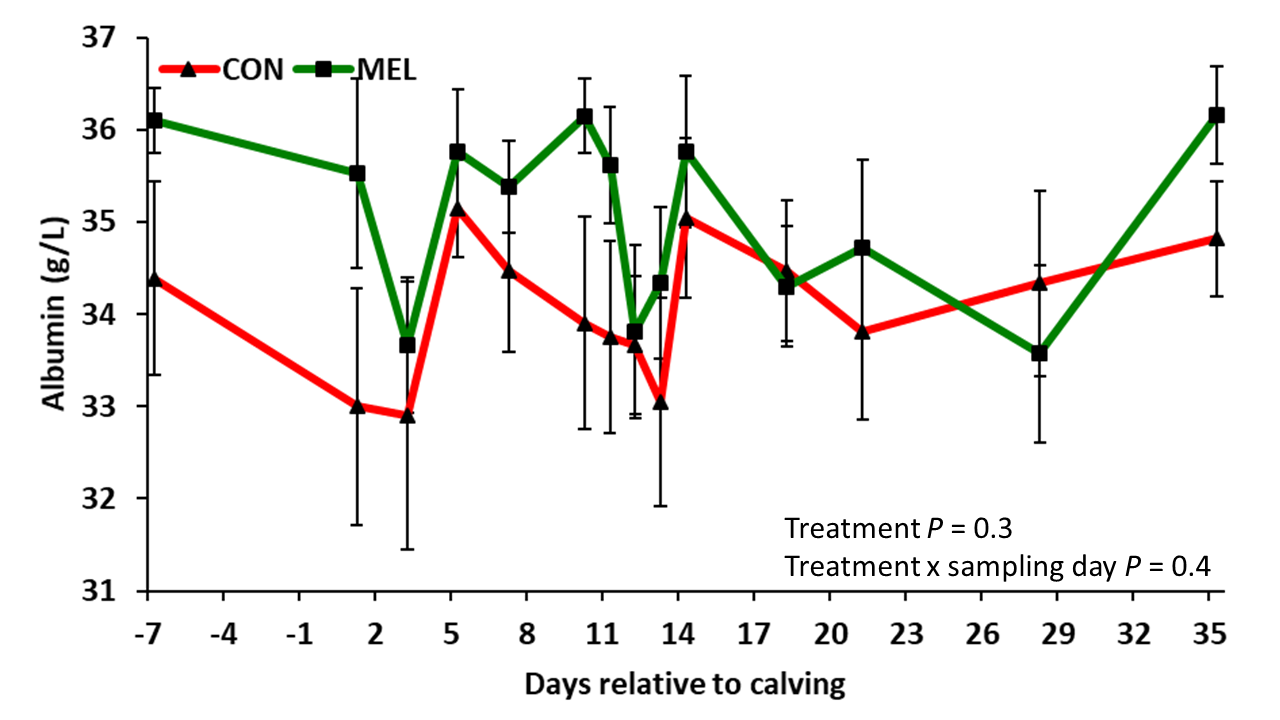


**Supplemental Figure 6.** LSM (accounting for parity and body condition score) ± SE of serum albumin concentrations in 42 Holstein cows. Experimental groups consisted of control (CON; n = 22) and meloxicam treated cows (MEL, n = 20). MEL received meloxicam (0.5 of mg/kg body weight) once daily for 4 days (10 to 13 days postpartum). There were no differences between experimental groups.


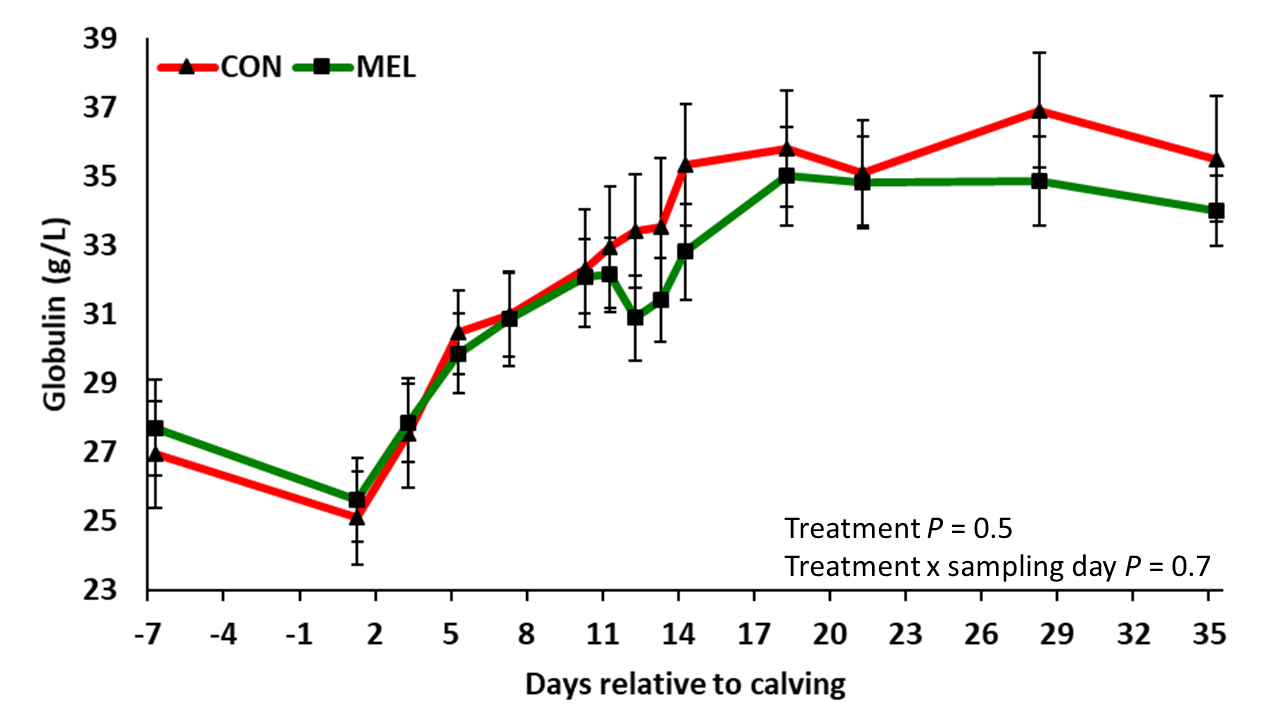


**Supplemental Figure 7.** LSM (accounting for parity and body condition score) ± SE of serum globulin concentrations in 42 Holstein cows. Experimental groups consisted of control (CON; n = 22) and meloxicam treated cows (MEL, n = 20). MEL received meloxicam (0.5 of mg/kg body weight) once daily for 4 days (10 to 13 days postpartum). There were no differences between experimental groups.


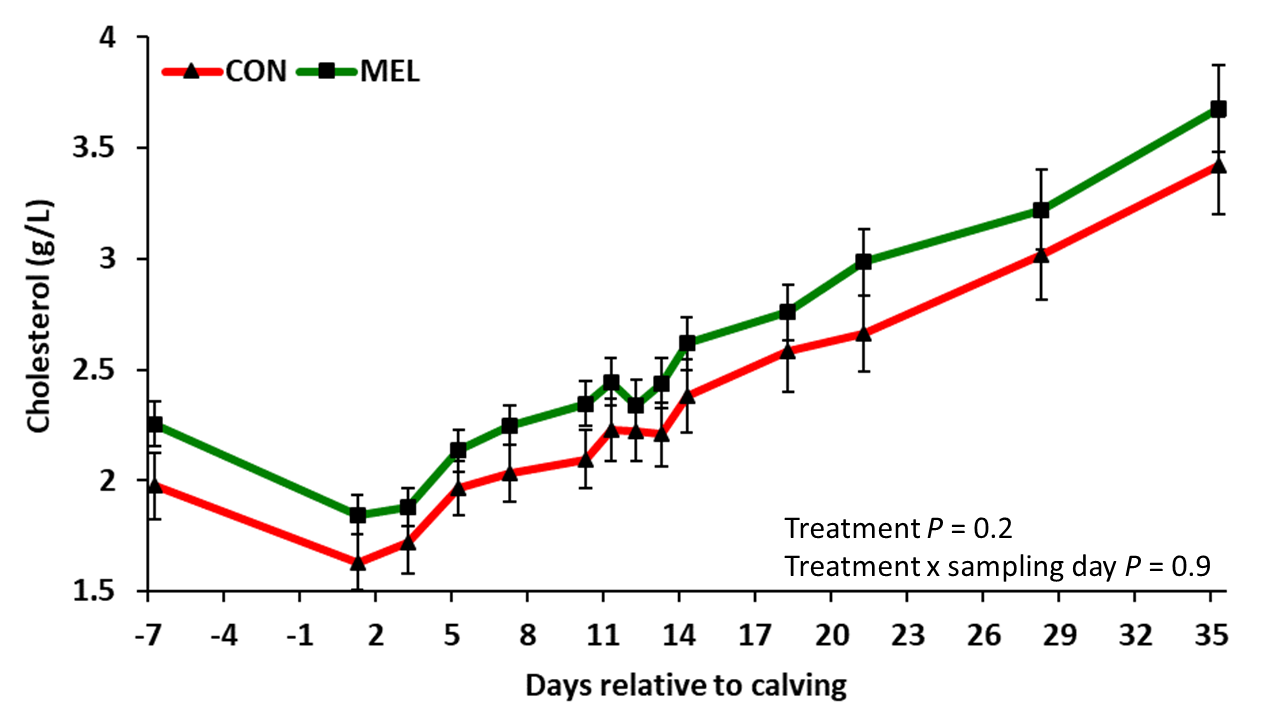


**Supplemental Figure 8.** LSM (accounting for parity and body condition score) ± SE of serum cholesterol concentrations in 42 Holstein cows. Experimental groups consisted of control (CON; n = 22) and meloxicam treated cows (MEL, n = 20). MEL received meloxicam (0.5 of mg/kg body weight) once daily for 4 days (10 to 13 days postpartum). There were no differences between experimental groups.


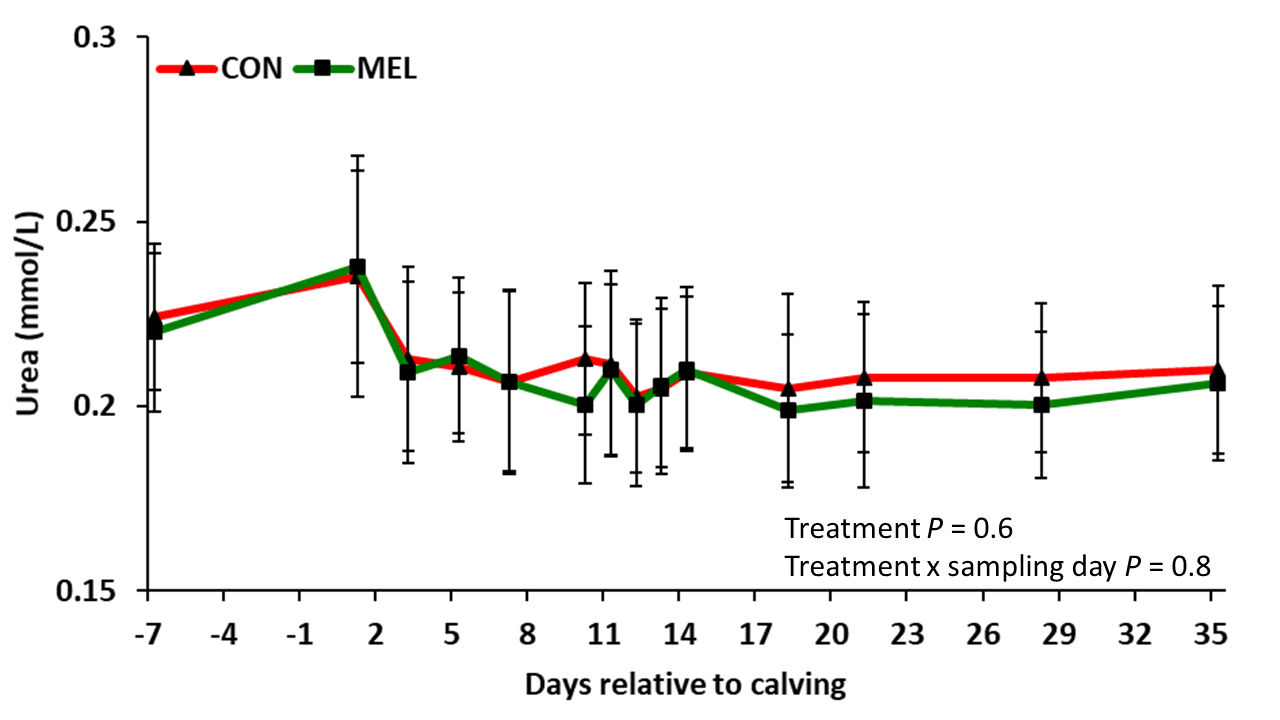


**Supplemental Figure 9.** LSM (accounting for parity and body condition score) ± SE of serum urea concentrations in 42 Holstein cows. Experimental groups consisted of control (CON; n = 22) and meloxicam treated cows (MEL, n = 20). MEL received meloxicam (0.5 of mg/kg body weight) once daily for 4 days (10 to 13 days postpartum). There were no differences between experimental groups.


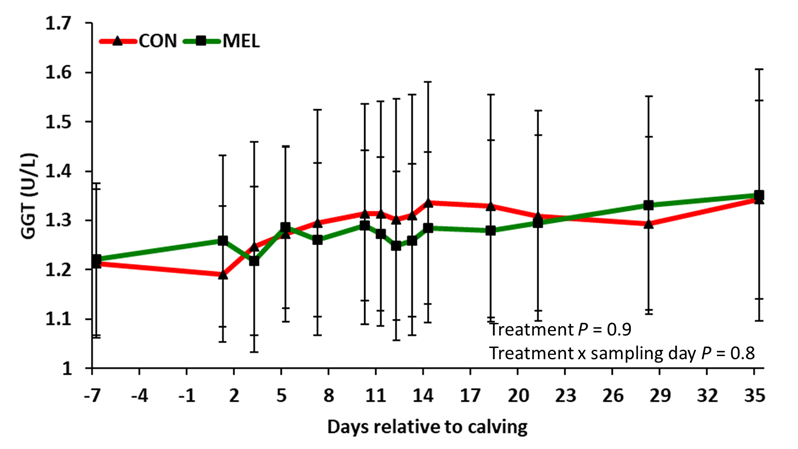


**Supplemental Figure 10.** Log_10_-scale LSM (accounting for parity and body condition score) ± SE of serum gamma-glutamyl transferase (GGT) concentrations in 42 Holstein cows. Experimental groups consisted of control (CON; n = 22) and meloxicam treated cows (MEL, n = 20). MEL received meloxicam (0.5 mg/kg of body weight) once daily for 4 days (10 to 13 days postpartum). There were no differences between experimental groups.


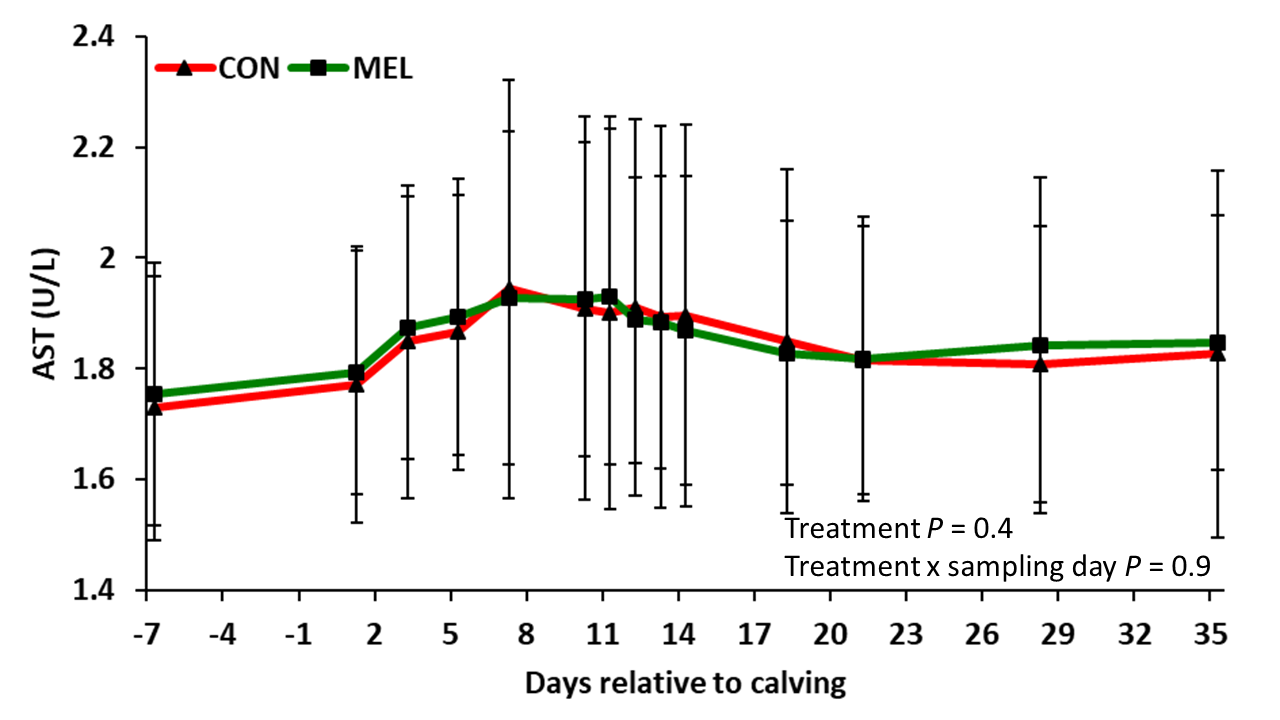


**Supplemental Figure 11.** Log_10_-scale LSM (accounting for parity and body condition score) ± SE of serum aspartate aminotransferase (AST) concentrations in 42 Holstein cows. Experimental groups consisted of control (CON; n = 22) and meloxicam treated cows (MEL, n = 20). MEL received meloxicam (0.5 mg/kg of body weight) once daily for 4 days (10 to 13 days postpartum). There were no differences between experimental groups.


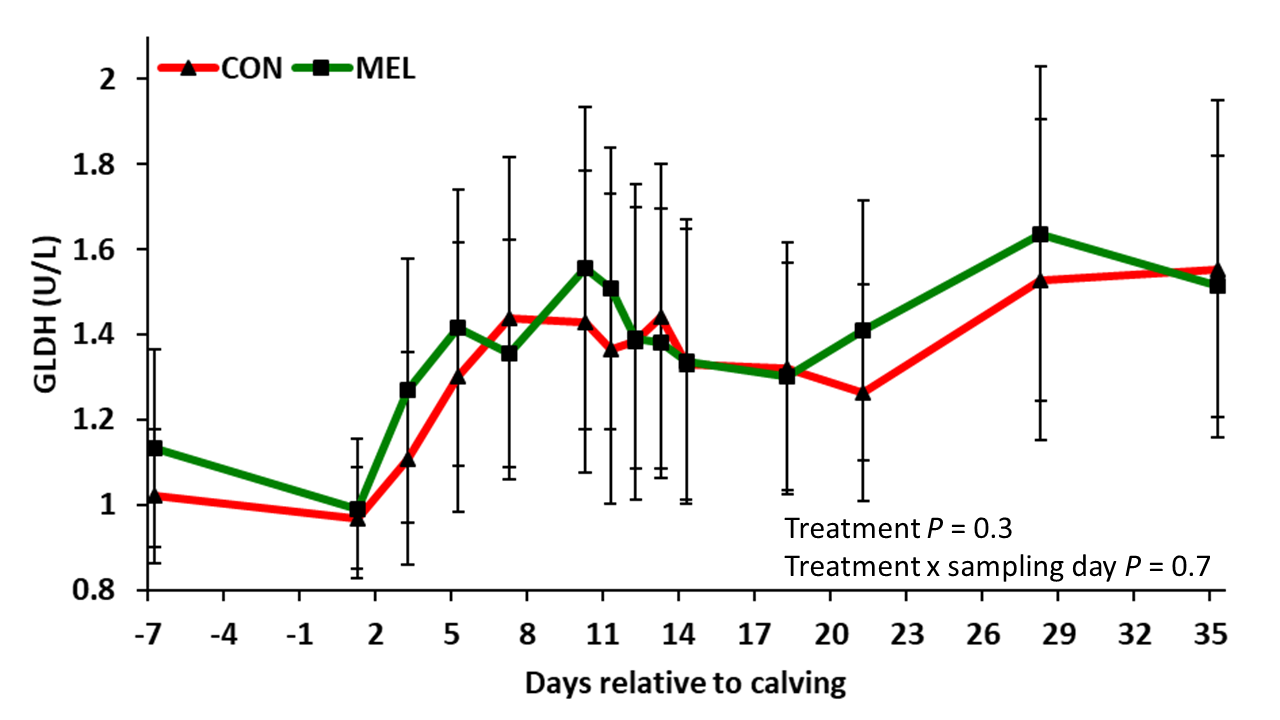


**Supplemental Figure 12.** Log_10_-scale LSM (accounting for parity and body condition score) ± SE of serum glutamate dehydrogenase (GLDH) concentrations in 42 Holstein cows. Experimental groups consisted of control (CON; n = 22) and meloxicam treated cows (MEL, n = 20). MEL received meloxicam (0.5 mg/kg of body weight) once daily for 4 days (10 to 13 days postpartum). There were no differences between experimental groups.


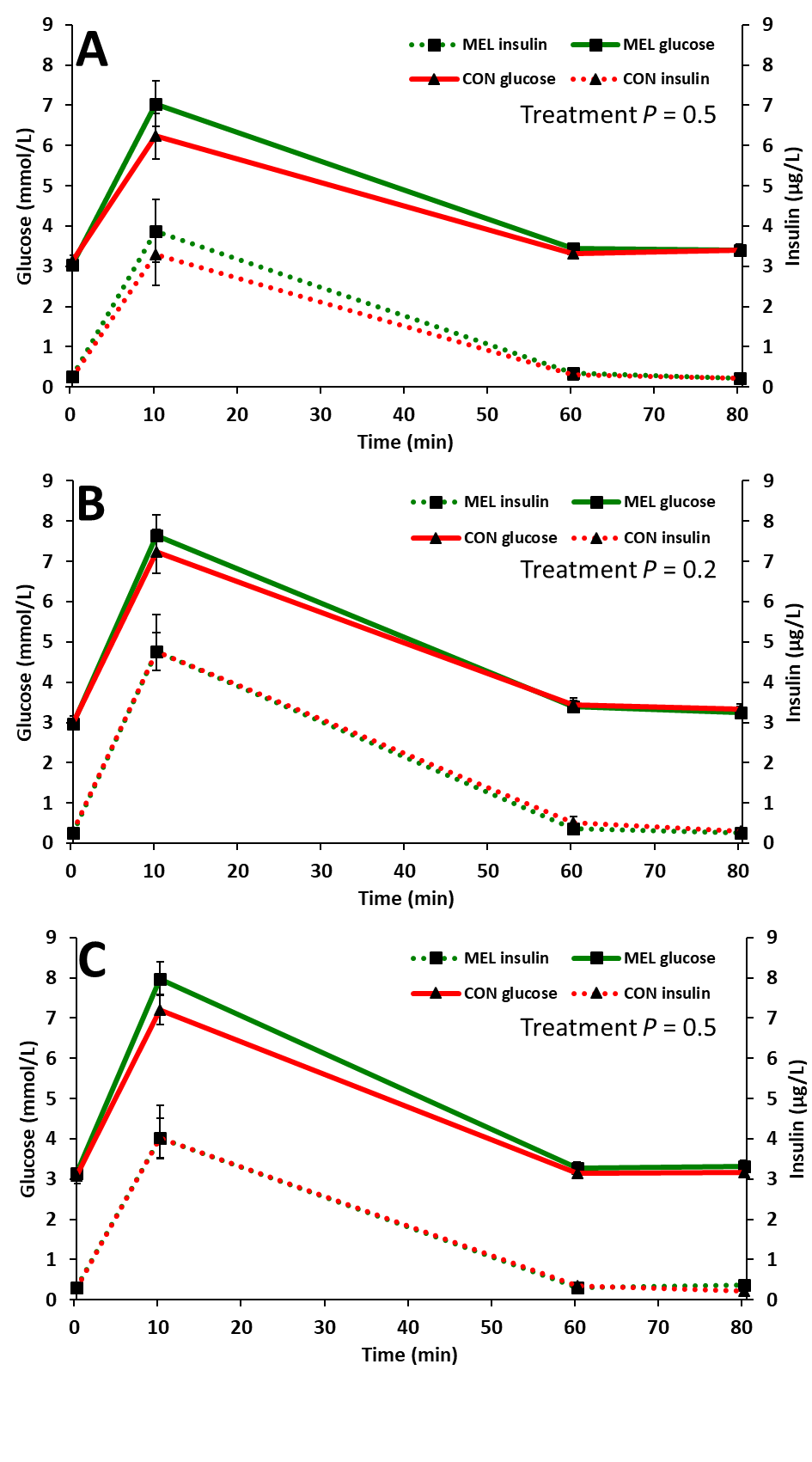


**Supplemental Figure 13.** LSM (accounting for parity and body condition score) ± SE of serum insulin and glucose concentrations of 42 Holstein cows. Experimental groups consisted of control (CON; n = 22) and meloxicam treated cows (MEL, n = 20). MEL received meloxicam (0.5 mg/kg of body weight) once daily for 4 days (10 to 13 days postpartum). These graphs depict results of simplified glucose tolerance tests performed 5, 10, and 14 days postpartum (A, B, and C figure, respectively). Glucose tolerance tests consisted of intravenous administration of 0.25 g of dextrose/kg of body weight over 2 min. A baseline blood sample (0 min) was collected immediately before dextrose infusion, and 10, 60, and 80 min following the dextrose infusion. No treatment effect on the area between the glucose and insulin curves was found at 5 (CON, 119.6 ± 21.1; MEL, 124.3 ± 23.6), 10 (CON, 118.1 ± 22.8; MEL, 123.3 ± 23.9), or 14 (CON, 119.6 ± 30.8; MEL, 137.8 ± 22.3) days postpartum.


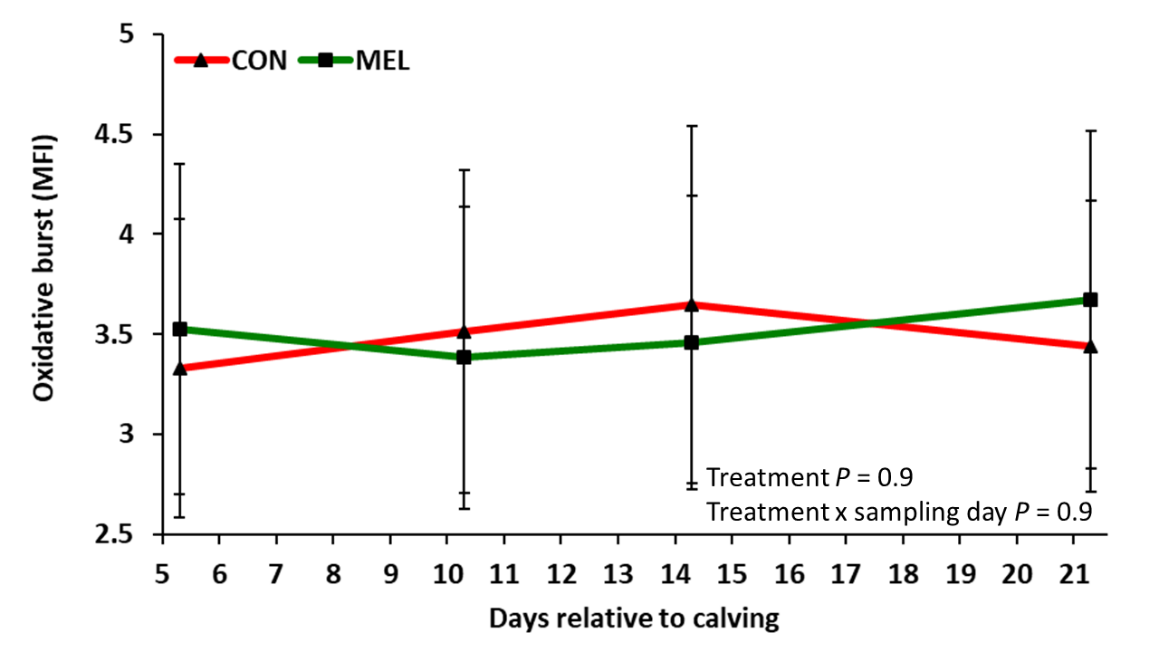


**Supplemental Figure 14.** Log_10_-scale LSM (accounting for parity and body condition score) ± SE of median fluorescence intensity (MFI) of phorbol myristate acetate-stimulated polymorphonuclear neutrophils relative to a negative control in 42 Holstein cows. Experimental groups consisted of control (CON; n = 22) and meloxicam treated cows (MEL, n = 20). MEL received meloxicam (0.5 mg/kg of body weight) once daily for 4 days (10 to 13 days postpartum). There were no differences between experimental groups.


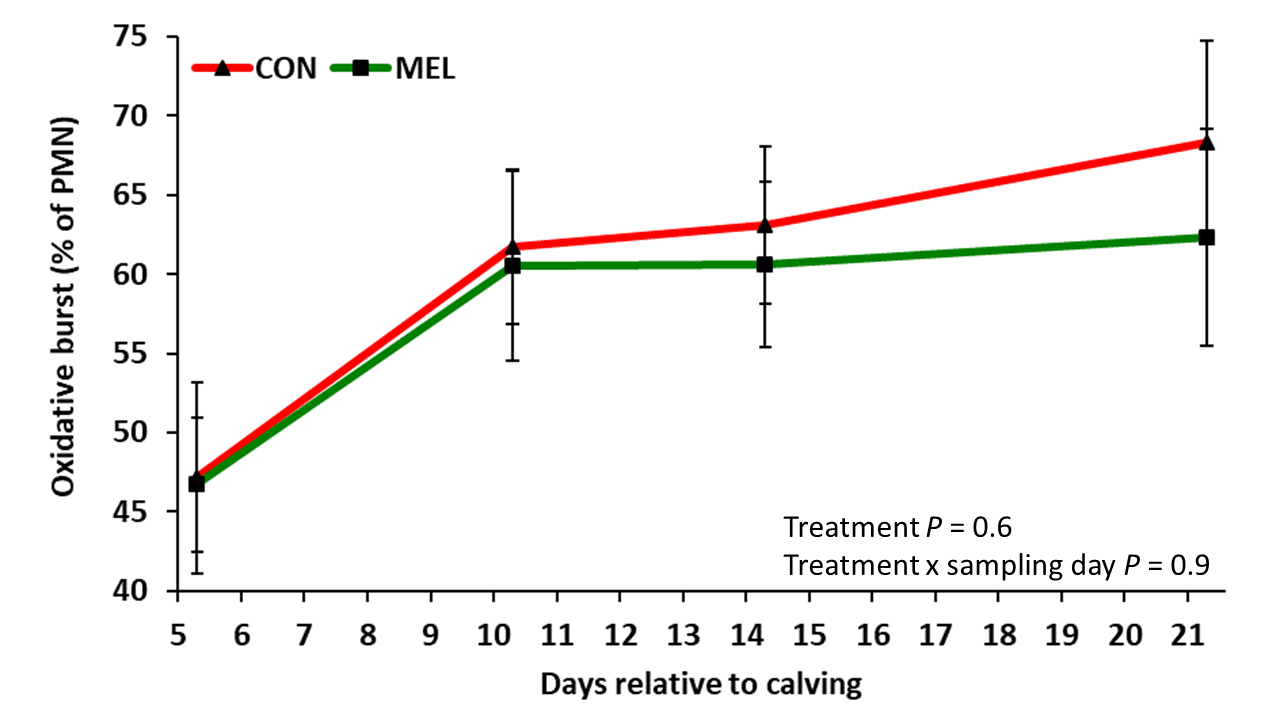


**Supplemental Figure 15.** LSM (accounting for parity and body condition score) ± SE of the shift in the percentage of phorbol myristate acetate stimulated polymorphonuclear neutrophils (PMN) relative to a negative control in 42 Holstein cows. Experimental groups consisted of control (CON; n = 22) and meloxicam treated cows (MEL, n = 20). MEL received meloxicam (0.5 mg/kg of body weight) once daily for 4 days (10 to 13 days postpartum). There were no differences between experimental groups.


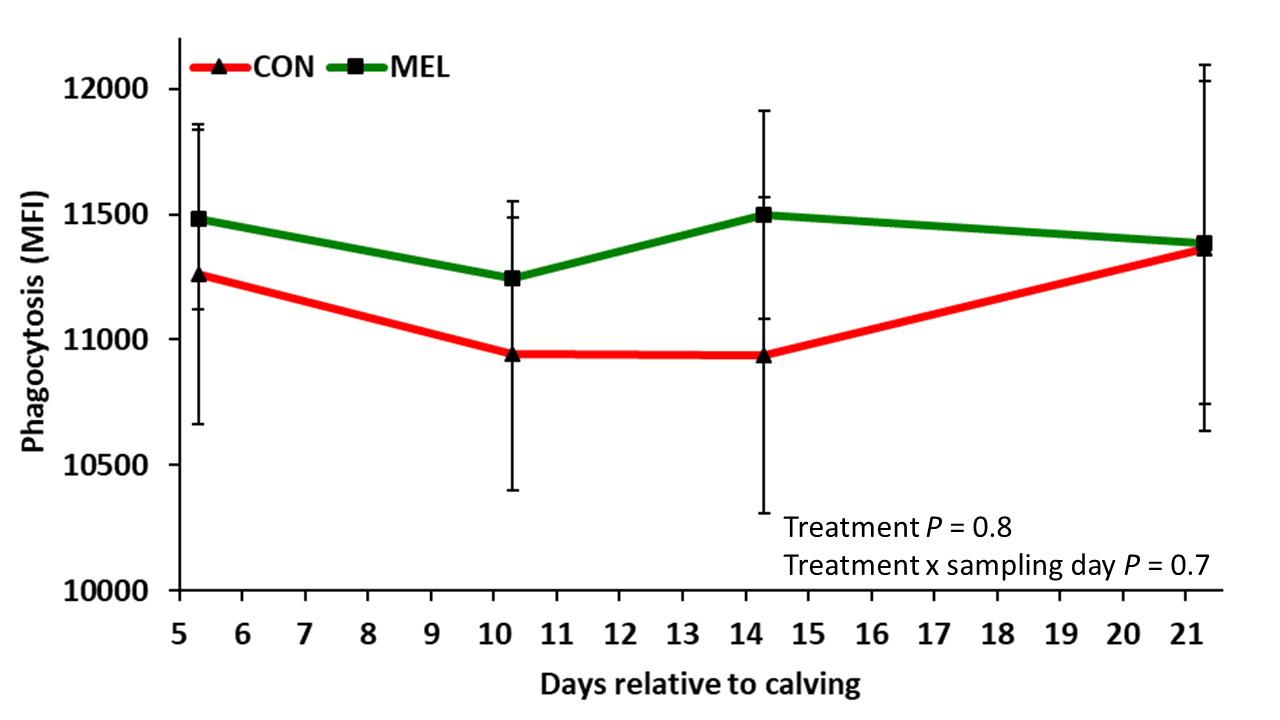


**Supplemental Figure 16.** LSM (accounting for parity and body condition score) ± SE of median fluorescence intensity (MFI) after fluorescent beads phagocytosis in 42 Holstein cows. MEL received meloxicam (0.5 mg/kg of body weight) once daily for 4 days (10 to 13 days postpartum). There were no differences between experimental groups.


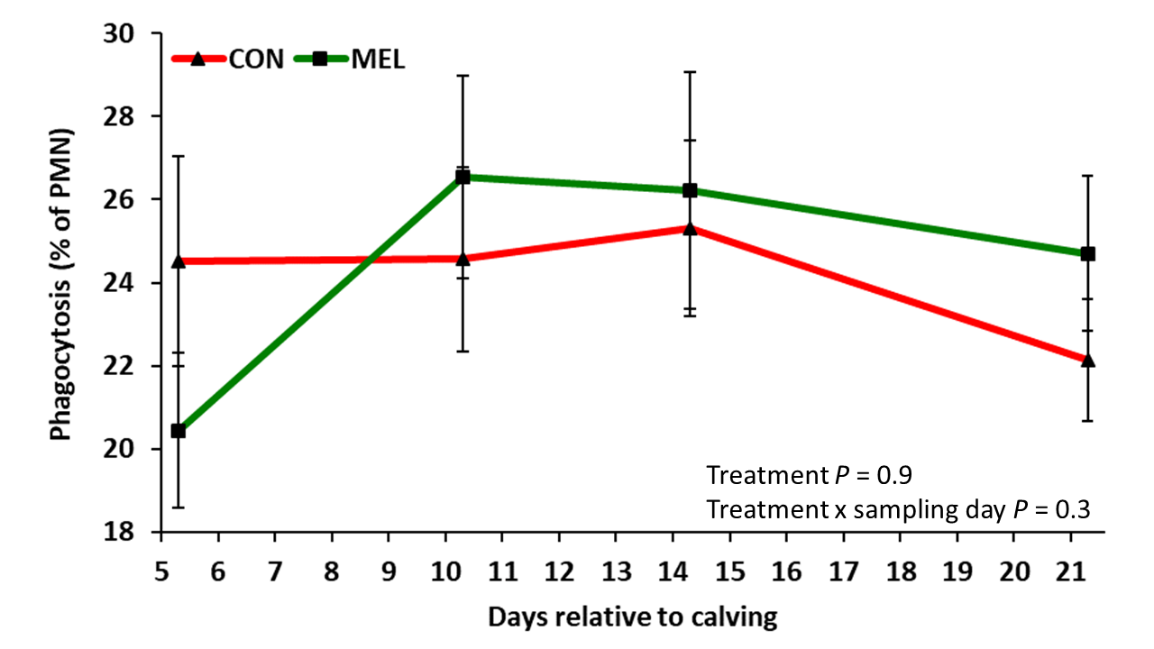


**Supplemental Figure 17.** LSM (accounting for parity and body condition score) ± SE of the percentage of polymorphonuclear neutrophils (PMN) that phagocyted fluorescent beads in 42 Holstein cows. Experimental groups consisted of control (CON; n = 22) and meloxicam treated cows (MEL, n = 20). MEL received meloxicam (0.5 mg/kg of body weight) once daily for 4 days (10 to 13 days postpartum). MEL tended to have greater percentage of PMN that phagocytosed fluorescent beads when compared to CON at 21 days postpartum (P = 0.09).
